# Life Stage-Specific Cargo Receptors Facilitate Glycosylphosphatidylinositol-Anchored Surface Coat Protein Transport in *Trypanosoma brucei*

**DOI:** 10.1128/mSphere.00282-17

**Published:** 2017-07-12

**Authors:** Emilia K. Kruzel, George P. Zimmett, James D. Bangs

**Affiliations:** Department of Microbiology and Immunology, University at Buffalo School of Medicine, Buffalo, New York, USA; Carnegie Mellon University

**Keywords:** COPII, ER exit, GPI anchor, *Trypanosoma*, cargo receptor, p24

## Abstract

The critical virulence factor of bloodstream-form *Trypanosoma brucei* is the glycosylphosphatidylinositol (GPI)-anchored variant surface glycoprotein (VSG). Endoplasmic reticulum (ER) exit of VSG is GPI dependent and relies on a discrete subset of COPII machinery (TbSec23.2/TbSec24.1). In other systems, p24 transmembrane adaptor proteins selectively recruit GPI-anchored cargo into nascent COPII vesicles. Trypanosomes have eight putative p24s (TbERP1 to TbERP8) that are constitutively expressed at the mRNA level. However, only four TbERP proteins (TbERP1, -2, -3, and -8) are detectable in bloodstream-form parasites. All four colocalize to ER exit sites, are required for efficient GPI-dependent ER exit, and are interdependent for steady-state stability. These results suggest shared function as an oligomeric ER GPI-cargo receptor. This cohort also mediates rapid forward trafficking of the soluble lysosomal hydrolase TbCatL. Procyclic insect-stage trypanosomes have a distinct surface protein, procyclin, bearing a different GPI anchor structure. A separate cohort of TbERP proteins (TbERP1, -2, -4, and -8) are expressed in procyclic parasites and also function in GPI-dependent ER exit. Collectively, these results suggest developmentally regulated TbERP cohorts, likely in obligate assemblies, that may recognize stage-specific GPI anchors to facilitate GPI-cargo trafficking throughout the parasite life cycle.

**IMPORTANCE** African trypanosomes are protozoan parasites that cause African sleeping sickness. Critical to the success of the parasite is the variant surface glycoprotein (VSG), which covers the parasite cell surface and which is essential for evasion of the host immune system. VSG is membrane bound by a glycolipid (GPI) anchor that is attached in the earliest compartment of the secretory pathway, the endoplasmic reticulum (ER). We have previously shown that the anchor acts as a positive forward trafficking signal for ER exit, implying a cognate receptor mechanism for GPI recognition and loading in coated cargo vesicles leaving the ER. Here, we characterize a family of small transmembrane proteins that act at adaptors for this process. This work adds to our understanding of general GPI function in eukaryotic cells and specifically in the synthesis and transport of the critical virulence factor of pathogenic African trypanosomes.

## Abstract (precis)

African trypanosomes are protozoan parasites that cause African sleeping sickness. Critical to the success of the parasite is the variant surface glycoprotein (VSG), which covers the parasite cell surface and which is essential for evasion of the host immune system. VSG is membrane bound by a glycolipid (GPI) anchor that is attached in the earliest compartment of the secretory pathway, the endoplasmic reticulum (ER). We have previously shown that the anchor acts as a positive forward trafficking signal for ER exit, implying a cognate receptor mechanism for GPI recognition and loading in coated cargo vesicles leaving the ER. Here, we characterize a family of small transmembrane proteins that act at adaptors for this process. This work adds to our understanding of general GPI function in eukaryotic cells and specifically in the synthesis and transport of the critical virulence factor of pathogenic African trypanosomes.

## INTRODUCTION

The protozoan parasite *Trypanosoma brucei* is the causative agent of African sleeping sickness as well as the related veterinary disease nagana. *T. brucei* is transmitted by the tsetse fly (*Glossina*) vector and has a life cycle of alternating growth forms specifically adapted to the conditions of either the mammalian bloodstream (bloodstream form [BSF]) or various niches within the tsetse fly (procyclic form [PCF] and others). Both BSF and PCF stages elaborate an extracellular glycocalyx composed of 5 to 10 million copies of specific coat proteins tethered to the plasma membrane by glycosylphosphatidylinositol (GPI) anchors. In BSF parasites, which grow extracellularly within the vasculature and tissue spaces and which consequently are directly exposed to host immunity, the variant surface glycoprotein (VSG) presents a homogenous antigenic surface that functions as a protective macromolecular barrier for invariant surface proteins below (1, 2). At a high frequency (10^−2^ to 10^−4^ per generation), the parasite stochastically switches its expressed VSG to an antigenically distinct one from among a repertoire of hundreds of genes stored throughout the genome (3, 4). This process, termed “antigenic variation,” is critical to pathogenesis and allows the parasite to continuously evade acquired host immunity (5, 6). VSG biosynthesis and presentation on the cell surface are thus essential to parasite success. In PCF cells, the surface coat is composed of a limited repertoire of procyclins (EP1, EP2, EP3, and GPEET) tethered by a distinct GPI anchor structure (7). This coat creates a highly polyanionic surface that aids in resistance to the harsh proteolytic environment of the tsetse fly midgut (8). Thus, robust GPI-anchored surface coats and the capacity to change them in response to environmental conditions are critical to trypanosome transmission and pathogenesis. However, despite this crucial role of GPI-cargo proteins throughout the parasite life cycle, the endogenous machinery involved in the manufacturing and transport of these critical virulence factors remains poorly characterized.

After attachment to nascent secretory cargo proteins in the endoplasmic reticulum (ER), GPI anchors function as a forward trafficking signal in both BSF and PCF cells. In BSF trypanosomes, GPI-minus VSG is severely delayed in ER exit, leading to accumulation and eventual misdirection in post-Golgi compartments from the cell surface toward the lysosome (9). Conversely, transport of BiPN, a small globular bulk flow secretory reporter based on the endogenous ER chaperone BiP, from the ER is accelerated by the addition of a GPI attachment sequence (BiPN:GPI) (10). Similarly, in PCF cells, GPI anchors on secretory reporter molecules, including ectopically expressed VSG, accelerate transport kinetics (11, 12). Combined, these data indicate that the GPI anchor alone is a forward trafficking signal in the early secretory pathway of trypanosomes.

ER exit in all eukaryotes is mediated by coat protein II (COPII)-coated vesicles that bud from distinct ribosome-free regions of the ER membrane known as ER exit sites (ERES) (13). COPII vesicle formation at ERES is initiated by the activation of the Sar1 GTPase, which embeds into the cytoplasmic face of the ER membrane and recruits Sec23/Sec24 heterodimers in a cage-like structure (14, 15). Subsequently, Sec13/Sec31 heterotetramers assemble on the prebudded complex and trigger membrane deformation and vesicle scission from the ER (15–17). Assembly of COPII machinery on the cytosolic face of the membrane simultaneously promotes the selective recruitment of transmembrane secretory cargoes via direct interactions between motifs in their cytosolic regions with Sec24 (18–20). For other secreted proteins, including both soluble and GPI-anchored proteins, which cannot interact directly with the COPII machinery (21), it is widely presumed that transmembrane protein adaptors facilitate cargo loading during COPII coat assembly.

*T. brucei* expresses two distinct, obligate, Sec23/24 heterodimers: TbSec23.1/TbSec24.2 and TbSec23.2/TbSec24.1 (22). Whether the two distinct heterodimers form separate homotypic classes of COPII vesicles or are included in a single heterotypic class is not known. In either case, only TbSec23.2/TbSec24.1 is required for efficient VSG exit from the ER. The same is true for other GPI-anchored cargoes, such as the abovementioned BiPN:GPI reporter. Since these cargoes reside in the inner leaflet of the ER membrane and cannot interact directly with the cytosolic COPII subunits, a transmembrane cargo receptor must serve as an adaptor to facilitate recruitment to specific TbSec23.2/TbSec24.1-mediated vesicle budding events.

GPI-dependent ER exit has been described in both yeast and mammalian cells and relies on members of the p24 family of transmembrane proteins. p24 proteins were originally identified as abundant 21- to 27-kDa proteins in small vesicles isolated from yeast (23). They are type I transmembrane proteins exhibiting a conserved domain architecture: a large, N-terminal GOLD (Golgi dynamics) domain with an adjacent coiled-coil region (predicted for oligomerization), a single transmembrane domain, and a short C-terminal tail. This cytoplasmic domain contains peptide motifs for interaction with COPII for ER exit (dihydrophobic) and for interaction with COPI [coat protein I, K(X)KXX] for retrograde Golgi compartment-to-ER transport (24). Thus, p24s facilitate ER-to-Golgi compartment transport of secretory cargo and then recycle back to the ER for further rounds of cargo transport. p24 proteins are predicted to assemble into heterooligomeric complexes and are also implicated in wide-ranging cellular functions beyond cargo loading, including ER quality control, membrane lipid dynamics, and the unfolded protein response (25–29). Due to the large size of p24 gene families in eukaryotic genomes (8 in *Saccharomyces cerevisiae*, 11 in *Arabidopsis thaliana*), it is widely presumed that the combinatorial interactions among p24 subunits generate higher-order complexes with various ligand-binding specificities, subcellular localizations, and biological roles, although supporting data are scarce. The cargo binding specificities of only two p24 complexes, one in yeast and one in mammalian cells, have been formally described. Both are comprised of at least four p24 subunits, and the resulting transmembrane complex interacts directly with the GPI anchor glycan to promote the inclusion of GPI-anchored proteins into COPII vesicles (30, 31).

Given that VSG transport out of the ER depends on GPI structures and a specific subset of COPII machinery, we hypothesize that p24 orthologues in *T. brucei* may serve as adaptors to facilitate VSG incorporation into COPII vesicles. We have identified eight putative p24 orthologues in the *T. brucei* genome that we refer to as TbERP1 to TbERP8 (*T. brucei* Emp24-related protein 1 to 8). In this work, we present a genetic characterization of these candidates using RNA interference (RNAi) silencing and transport assays for multiple endogenous and recombinant secretory cargoes. We have found that a subset is expressed during bloodstream-form growth: TbERP1, TbERP2, TbERP3, and TbERP8. They are required for GPI-dependent transport, localize to ER exit sites (ERES), and appear to assemble into higher-order oligomeric complexes. A distinct subset (TbERP1, TbERP2, TbERP4, and TbERP8) is expressed in procyclic-form cells, which synthesize different GPI structures. Our results suggest that regulated TbERP cohorts, likely assembled into obligate complexes, recognize stage-specific GPI anchors to facilitate the transport of GPI cargo out of the ER throughout the parasite life cycle.

## RESULTS

### Identification of trypanosomal p24 genes.

The *T. brucei* genome database (http://tritrypdb.org/tritrypdb/) was queried with the protein sequences of known members of the p24 family (Emp24p, YGL200C; Erv25, YML012W) from *S. cerevisiae*. Eight putative p24 orthologues were identified by reciprocal BLAST analyses (see Table S1 in the supplemental material). These are referred to here as TbERP1 to TbERP8 (*T. brucei* Emp24-related protein). The deduced TbERP proteins range in size from 203 to 253 amino acids in length and exhibit the classic conserved architecture of the p24 family (N to C): signal peptide, luminal GOLD domain, a heptad repeat region, a single transmembrane domain, and a short cytoplasmic tail (10 to 15 amino acids) with predicted COPI and COPII interaction motifs [K(X)KXX and dihydrophobic, respectively] (24).

10.1128/mSphere.00282-17.3 TABLE S1  *Trypanosoma brucei* p24 orthologues. Download TABLE S1, PDF file, 0.1 MB. Copyright © 2017 Kruzel et al. 2017 Kruzel et al. This content is distributed under the terms of the Creative Commons Attribution 4.0 International license.

### TbERP1, TbERP2, TbERP3, and TbERP8 localize to ER exit sites in BSF cells.

To investigate the localizations and functions of TbERP1 to TbERP8, we generated epitope-tagged BSF cell lines corresponding to TbERP1 to TbERP8. Both alleles of the TbERP genes were *in situ* tagged at their endogenous loci in order to maintain expression levels at or near wild-type levels. Due to the role of the short p24 C-terminal cytoplasmic domain in proper localization and function in other systems (30, 32), we opted for N-terminal placement of either hemagglutinin (HA) or TY tags immediately downstream of a signal sequence as described in Materials and Methods. The fusion proteins were first assayed for stable expression and appropriate size by Western blotting with anti-TY or anti-HA antibodies. Epitope-tagged TbERP1, TbERP2, TbERP3, and TbERP8 were readily detectable by immunoblotting (Fig. S1A). TbERP4, TbERP5, TbERP6, and TbERP7, however, were undetectable, even when both endogenous alleles were tagged. To eliminate the possibility that the epitope tags themselves were destabilizing to TbERP4 to TbERP7, we overexpressed TY-tagged TbERP4 to TbERP7 using a tetracycline-inducible vector. When induced, these proteins were detectable by immunoblotting at the appropriate sizes (not shown), indicating that the epitope tags are not inherently destabilizing. mRNAs for all eight TbERPs, however, were readily detected by quantitative real-time PCR (qRT-PCR) (Fig. S2). These results suggest posttranscriptional regulation of the TbERP cohort, resulting in the expression of TbERP1, TbERP2, TbERP3, and TbERP8 and the nonexpression of TbERP4 to TbERP7 proteins during BSF growth.

10.1128/mSphere.00282-17.1 FIG S1  TbERP knockdown and detection by Western blotting. (A) Total extracts of control (wild-type [WT]) and epitope-tagged cell lines were fractionated by SDS-PAGE (1 × 10^7^ cell equivalents per lane), immunoblotted with anti-TY (left panel), and then stripped/reprobed with anti-HA antibody (right panel). Molecular mass markers are indicated in kilodaltons. (Top) Lysates from BSF cells. (Bottom) Lysates from PCF cells. (B) BSF (left) and PCF (right) cell lines containing tetracycline-inducible TbERP RNAi constructs were cultured with or without tetracycline to initiate specific dsRNA production. Cells were evaluated after 72 h (BSF) or 48 h (PCF) by qRT-PCR to determine relative TbERP mRNA levels under knockdown using the internal control mRNA target ZFP3 (68). Mean ± SEM values from *n* = 3 biological replicates are presented. Download FIG S1, PDF file, 0.3 MB. Copyright © 2017 Kruzel et al. 2017 Kruzel et al. This content is distributed under the terms of the Creative Commons Attribution 4.0 International license.

10.1128/mSphere.00282-17.2 FIG S2  TbERP mRNA expression in BSF and PCF trypanosomes. BSF cells (expressing VSG221) and PCF cells (expressing EP procyclin) were evaluated for the expression of native TbERP mRNAs by qRT-PCR. cDNA was synthesized with (+RT) or without (-RT) reverse transcriptase and used as the template for qPCRs (*n* = 3 technical replicates). The mean cycle threshold (*C*_*T*_) value of the -RT samples was subtracted from the mean *C*_*T*_ value of the +RT samples, resulting in the Δ*C*_*T*_ value. Δ*C*_*T*_ values exceeding 2.0 were considered to demonstrate mRNA levels significantly above background. VSG221 and EP procyclin were used as positive controls for expression in BSF and PCF stages, respectively. VSG117 was used as a negative control for expression in both stages. Download FIG S2, PDF file, 0.1 MB. Copyright © 2017 Kruzel et al. 2017 Kruzel et al. This content is distributed under the terms of the Creative Commons Attribution 4.0 International license.

Before evaluating TbERP1, TbERP2, TbERP3, and TbERP8 localization, we verified that epitope tagging did not affect normal TbERP function. RNAi silencing experiments for each of these TbERPs indicated that they are required for efficient ER exit of lysosomal cathepsin L (TbCatL; see below). Trafficking of TbCatL in the tagged TbERP cell lines was found to be normal (data not shown). Since in each case both TbERP alleles were tagged, these results confirm that the tagged TbERPs are functional and thus can be used to evaluate endogenous subcellular localizations.

**FIG 1:**
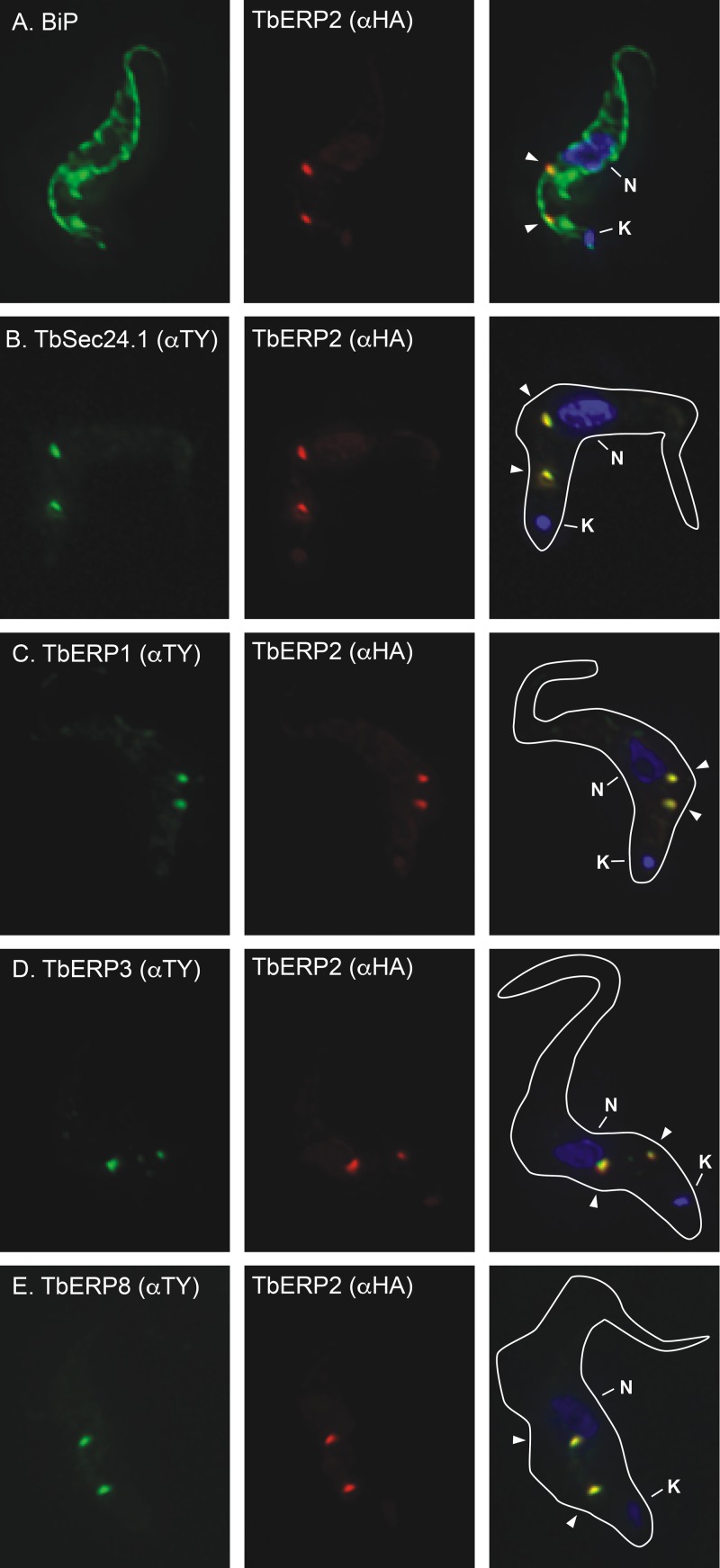
TbERP1, TbERP2, TbERP3, and TbERP8 colocalize at ER exit sites. The *in situ* TY tagging constructs for TbSec24.1 (B), TbERP1 (C), TbERP3 (D), and TbERP8 (E) were each transfected into the BSF HA-TbERP2 cell line (A), generating a panel of differentially tagged cell lines as indicated. The various cell lines were fixed, permeabilized, and stained with anti-BiP or anti-TY (left, as indicated) and anti-HA (middle). Cells were then stained with appropriate secondary antibodies (HA, red; BiP or TY, green) and DAPI to visualize nucleic acid (blue). Three-channel merged images and cell body outlines from corresponding DIC images are presented (right). All images are deconvolved sum-stack projections of representative G_1_-phase cells. Arrowheads, TbERP-positive foci; N, nucleus; K, kinetoplast.

The HA-TbERP2 cell line was costained for the ER chaperone BiP. Whereas characteristic ER staining can be seen throughout the cell body (anti-BiP), TbERP2 localizes to two distinct ER foci, between the nucleus and kinetoplast and adjacent to the flagellar adherent zone, in interphase cells (Fig. 1A; differential interference contrast [DIC] image not shown). This staining pattern is reminiscent of the trypanosomal ERES, where COPII (TbSec23 and TbSec24) occupancy on the ER membrane typically indicates two sites of active vesicle budding in BSF trypanosomes (22). We next generated a doubly tagged HA-TbERP2/TY-TbSec24.1 cell line. TbERP2 precisely colocalized with TbSec24.1 at all foci observed (Fig. 1B), confirming these structures as bona fide ERES. We used the same strategy to generate doubly tagged cell lines with HA-TbERP2 versus TY-TbERP1, TY-TbERP3, or TY-TbERP8. The anti-TY staining, in each case, precisely colocalized with HA-TbERP2 to foci within the postnuclear region (Fig. 1C to E). These results conclusively demonstrate colocalization of TbERP1, TbERP2, TbERP3, and TbERP8 at ERES in BSF trypanosomes.

### TbERP silencing by RNAi.

In order to investigate the functions of TbERP1, TbERP2, TbERP3, and TbERP8 in BSF cells, we conditionally silenced each target independently via RNAi, using specific tetracycline-inducible stem-loop double-stranded RNA (dsRNA) constructs. After 72 h of dsRNA induction, cells were harvested and assessed for knockdown of target message using quantitative real-time PCR (qRT-PCR). Significant depletion of target message was achieved for TbERP1, TbERP2, TbERP3, and TbERP8 (depleted to 9.1%, 12.1%, 17.8%, and 6.9% of control mRNA levels, respectively; *n* = 3 biological replicates) (Fig. S1B). TbERP4, TbERP5, TbERP6, and TbERP7 mRNA levels were unaffected by induction of specific dsRNA (not shown).

**FIG 2:**
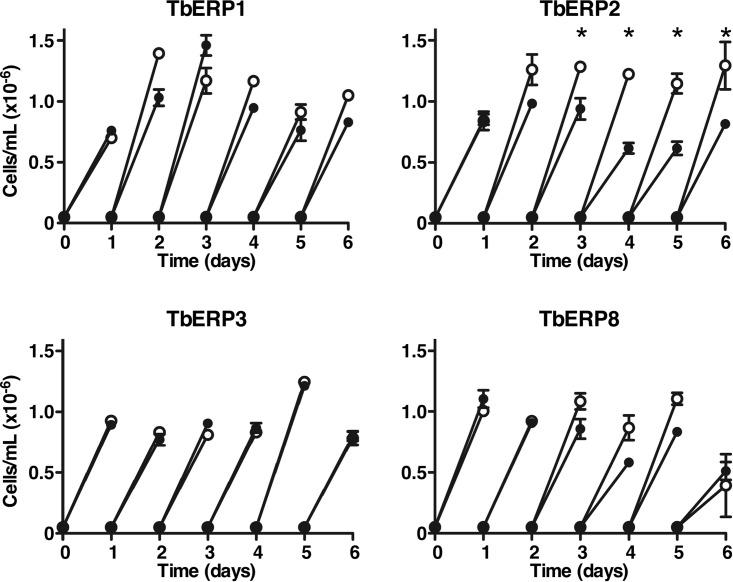
TbERP silencing has minimal effect on proliferation. Transgenic BSF cell lines expressing the tetracycline-inducible TbERP RNAi constructs were cultured with (filled circles) or without (open circles) tetracycline. Cell density was measured with a hemacytometer, and cells were adjusted back to the starting density every 24 h. Mean ± SEM values from triplicate cultures (for each treatment) are presented (*, *P* < 0.05, Student’s *t* test). TbERP2 silencing caused a modest growth defect (~50%), observable after 72 h of RNAi induction. All subsequent RNAi phenotypic analyses of TbERP1, TbERP2, TbERP3, and TbERP8 were performed at 72 h of RNAi induction in BSF cells.

Each RNAi cell line was evaluated for growth in the presence and absence of tetracycline to assess the effect of TbERP silencing on viability. Silencing of TbERP2 caused a modest but significant growth defect observable after 72 h of RNAi induction (Fig. 2). TbERP1, TbERP3, or TbERP8 silencing, however, had no effect on growth, even after 6 days of continuous silencing. This may be due to incomplete knockdown of target message, with residual mRNA sufficient to maintain wild-type growth and/or function. Alternatively, their roles may in fact be nonessential or redundant in BSF cells. Based on the TbERP2 results, all subsequent analyses were performed at 72 h of silencing.

### Secretory transport of VSG is TbERP dependent.

**FIG 3:**
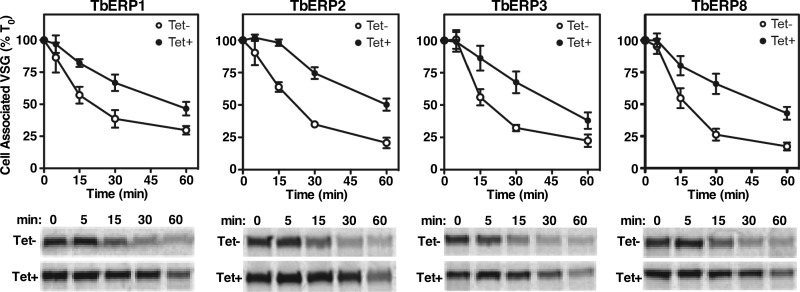
VSG trafficking is TbERP dependent. Specific dsRNA was induced for 72 h in each of the BSF TbERP RNAi cell lines as indicated. Control (Tet-, open circles) and induced (Tet+, closed circles) cells were pulse (2 min)-chase (60 min) radiolabeled, and VSG trafficking was analyzed over time by hypotonic lysis as described in Materials and Methods. VSG was specifically immunoprecipitated from cell fractions and analyzed by SDS-PAGE and phosphorimaging (2 × 10^6^ cell equivalents per lane). (Top) The means ± SEM from biological triplicate experiments are presented as the loss of cell-associated VSG normalized to *T*0. (Bottom) Representative phosphorimages of cell-associated VSG.

In other systems, p24 proteins have been shown to facilitate GPI-anchored cargo exit from the ER and accelerate forward trafficking (25, 28, 33, 34). To evaluate a role of the TbERPs in GPI-dependent trafficking, we monitored VSG transport kinetics under TbERP silencing by pulse-chase radiolabeling. Surface arrival can be detected by onset of susceptibility of VSG to release by endogenous GPI-specific phospholipase C following hypotonic lysis (35). Newly synthesized VSG, still in transit, remains intact and cell associated under these conditions and thus can be separated by centrifugation from the surface VSG released by GPI cleavage (soluble). Under specific silencing of TbERP1, TbERP2, TbERP3, or TbERP8, the rate of VSG trafficking was significantly decreased relative to control cells (Fig. 3; ~2-fold; half-life [*t*_1/2_], ~50 min versus ~20 to 25 min). In each case, this is seen as a prolonged retention of radiolabeled VSG in the cell-associated fraction upon RNAi induction. Published studies have established the *t*_1/2_ of VSG transport in wild-type cells at ~15 min, which was reproduced in our nontransfected parental cells (not shown) (35, 36). We attribute the modestly delayed transit in uninduced RNAi cells to leakiness of the RNAi constructs. Consequently, the observed RNAi effects are likely even greater than those with untransfected parental cells (3- to 4-fold). The phenocopying of the four TbERPs was surprising, given that only TbERP2 silencing caused an observable growth defect. This indicates that the delay in VSG transport seen in TbERP1, TbERP3, and TbERP8 RNAi, although significant, is insufficient alone to negatively affect proliferation and viability *in vitro*. These data also imply that TbERP2 may have roles beyond VSG trafficking that, in combination, manifest as the observed growth defect.

### A single GPI anchor confers TbERP-dependent trafficking.

**FIG 4:**
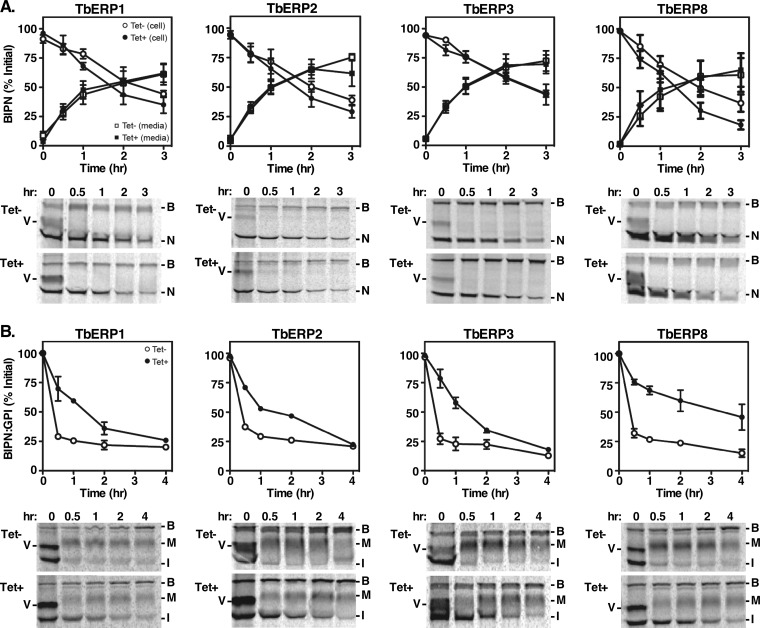
A GPI anchor is sufficient to confer TbERP-dependent trafficking to BiPN. The matched soluble BiPN (A) and BiPN:GPI (B) secretory reporters were each constitutively expressed in each BSF TbERP RNAi cell line as indicated. Specific dsRNA was induced for 72 h (Tet-, open symbols; Tet+, closed symbols). Cells were pulse (10-min)-chase (3 or 4 h) radiolabeled, and reporter trafficking was analyzed by specific immunoprecipitation (anti-BiP) and SDS-PAGE at the indicated chase times. BiPN was immunoprecipitated from both whole-cell lysates (circles) and medium fractions (squares); BiPN:GPI was immunoprecipitated from whole-cell lysates only (circles). BiPN trafficking was assessed by loss of cell-associated reporter and appearance in the medium; BiPN:GPI trafficking was assessed by conversion to the larger Golgi compartment-processed glycoform. (Top) Quantification of cell-associated (circles) and secreted (squares) reporters normalized to total initial signal at *T*0 (mean ± SEM, *n* = 3). (Bottom) Representative phosphorimages (1 × 10^7^ cell equivalents per lane). B, native BiP; V, VSG; N, BiPN; I, immature precursor BiPN:GPI; M, mature BiPN:GPI. Note that newly synthesized VSG coprecipitates with native BiP at *T*0 due to transient physical association during folding/assembly into homodimers (37).

To determine if the VSG trafficking defect is cargo specific, we expressed the constitutive BiPN secretory reporter and its GPI-anchored counterpart BiPN:GPI in each of the transgenic TbERP RNAi cell lines. BiPN is the N-terminal ATPase domain of the endogenous ER chaperone BiP (37). It lacks positive trafficking signals and thus serves as a bulk flow secretory reporter. It is secreted from BSF trypanosomes into the medium with established kinetics of a *t*_1/2_ of ~90 min (9, 37). We evaluated the rates of BiPN trafficking under specific RNAi against TbERP1, TbERP2, TbERP3, or TbERP8 by pulse-chase analysis and immunoprecipitation with anti-BiP antibody (Fig. 4A). Endogenous BiP (marked as B in Fig. 4) was immunoprecipitated in all samples, demonstrating equivalent loading. Newly synthesized VSG (V), in transient physical association with endogenous BiP, was also detected in all time zero samples, as seen previously (37). Depletion of TbERP1, TbERP2, TbERP3, or TbERP8 had no effect on the rates of BiPN (N) reporter secretion into the medium, indicating that TbERP depletion does not affect bulk secretion or otherwise compromise secretory function.

To assess the GPI dependence of the TbERP VSG phenotypes, we utilized BiPN:GPI, a modified version of the BiPN reporter that exhibits GPI-dependent accelerated trafficking relative to BiPN (10). Like VSG, its trafficking is also dependent on the TbSec23.2/TbSec24.1 heterodimer (22). BiPN:GPI is initially synthesized as a GPI-anchored ER glycoform (I, 55 kDa) and is converted to a larger mature glycoform (M) during post-ER transport, presumably by GPI anchor processing as no such processing occurs on GPI-minus BiPN. The kinetics of BiPN:GPI ER exit can thus be inferred from the rate of conversion of the immature form to the mature form. Silencing of TbERP1 severely delayed (~4.5-fold) BiPN:GPI transport relative to control cells (Fig. 4B, *t*_1/2_, ~90 min versus ~20 min). Importantly, the reduced rate closely mimics the kinetics of unmodified BiPN trafficking (*t*_1/2_, ~90 min), suggesting that TbERP1 is involved in most (if not all) of the GPI-dependent acceleration in BiPN transport. Silencing of TbERP3 or TbERP8 had very similar effects, suggesting comparable roles in GPI-dependent forward trafficking.

Evaluation of BiPN:GPI in the TbERP2 RNAi cells was not as straightforward. Upon RNAi induction, individual cultures frequently exhibited a severe growth defect. In such cultures, the BiPN:GPI reporter was undetectable by immunoblotting (not shown), suggestive of selection for reporter silencing. This phenomenon occurred stochastically during standard maintenance culture and was observed in four independent clonal cell lines (not shown). It is worth noting that TbERP2 RNAi cells did not exhibit this behavior when constitutively expressing the GPI-minus BiPN reporter (Fig. 4A). Nor has this phenomenon been observed in any other RNAi cell line in which BiPN:GPI has been assayed to date (10, 22, 38), including the TbERP1, TbERP3, and TbERP8 RNAi cell lines (this work). Thus, it appears that the TbERP2 RNAi cell line is uniquely sensitive to the overexpression of this particular GPI-anchored reporter construct. We interpret this puzzling result as a synergistic toxicity between TbERP2 depletion and constitutive BiPN:GPI overexpression. The TbERP2 RNAi cell line was thus technically challenging to assess quantitatively for BiPN:GPI transport rates. However, we did manage a single technically robust experiment evaluating BiPN:GPI trafficking after a shorter RNAi induction (20 h): BiPN:GPI trafficking was delayed 3-fold under this condition (Fig. 4B). Collectively, these data support a model whereby a single GPI anchor is sufficient to confer TbERP-dependent transport from the ER.

### Secretory transport of TbCatL is TbERP dependent.

**FIG 5:**
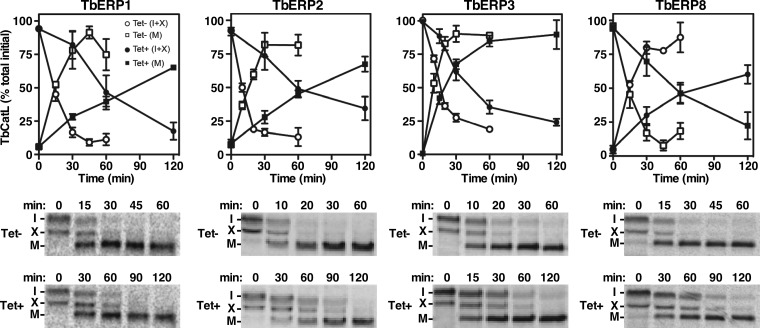
Biosynthetic trafficking of TbCatL is TbERP dependent. Specific dsRNA was induced for 72 h in each of the BSF TbERP RNAi cell lines as indicated. Control (Tet-, open symbols) and induced (Tet+, closed symbols) cells were pulse (10 min)-chase (2 h) radiolabeled, and TbCatL trafficking (conversion to the mature form) was analyzed over time as described in Materials and Methods. TbCatL was specifically immunoprecipitated from total cell lysates at the indicated chase times and analyzed by SDS-PAGE and phosphorimaging (1 × 10^7^ cell equivalents per lane). (Top) Quantification of TbCatL isoforms normalized to total initial TbCatL (mean ± SEM for *n* = 3). Squares, mature TbCatL (M); circles, immature TbCatL forms (I+X). (Bottom) Representative phosphorimages are presented. Tet^+^ cultures were sampled at later time points to allow for more complete conversion of TbCatL.

To more fully characterize the functions of TbERP1, TbERP2, TbERP3, and TbERP8, we investigated their roles in biosynthetic trafficking of endogenous cathepsin L (TbCatL), a soluble lysosomal hydrolase. TbCatL is initially synthesized in the ER as a mixture of a larger primary precursor (I, 53 kDa) and a smaller secondary immature precursor form (X, 50 kDa). These undergo proteolytic activation upon lysosomal arrival, resulting in a single high-mobility mature form (M, 44 kDa) (39). RNAi silencing of TbERP1, TbERP2, TbERP3, or TbERP8 all caused significant delays in TbCatL trafficking, observable by a delayed conversion to the mature form (M) in the induced cells (Fig. 5). The effect of TbERP1, TbERP2, or TbERP8 silencing was more severe (~4-fold) than that observed for TbERP3 (~2-fold).

### TbERP proteins are dependent on one another for stability.

In both yeast and mammalian cells, individual p24 proteins are stabilized by complex formation, and subunits are unstable in the absence (or depletion) of other subunits (25, 28, 31, 40). To determine the interdependencies of the TbERP proteins and the potential for higher-order complexes, we evaluated TbERP1, TbERP2, TbERP3, and TbERP8 levels following specific RNAi silencing of opposing TbERP genes. The epitope-tagged TbERP constructs (identical to those used in Fig. 1) were transfected into the tetracycline-inducible RNAi cell lines, and expression levels were monitored by tag-specific immunoblotting following 72 h of RNAi silencing.

**FIG 6:**
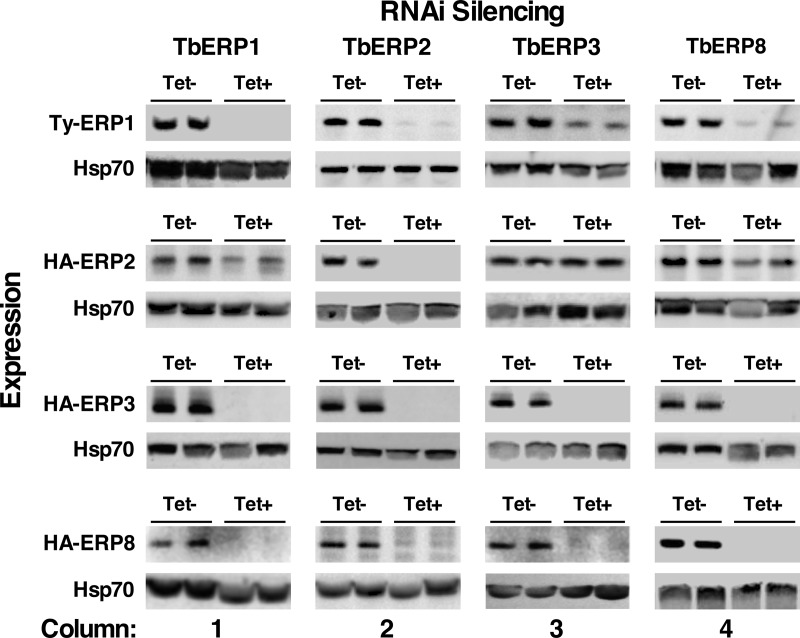
TbERP1, TbERP2, TbERP3, and TbERP8 are codependent for stability. The *in situ* TbERP epitope-tagging constructs were transfected into each of the TbERP RNAi cell lines. Specific dsRNA was induced for 72 h, and whole-cell lysates were prepared from control (Tet-) and induced (Tet+) cells, as indicated in the column labels. Lysates were fractionated by SDS-PAGE (1 × 10^7^ cell equivalents per lane). Membranes were first immunoblotted with anti-TY or anti-HA to evaluate TbERP protein stability under RNAi, as indicated in the row labels. Blots were then stripped and reprobed for cytosolic Hsp70 as a loading control. Two biological replicates are presented (total *n* = 3). qRT-PCR analyses confirmed that RNAi knockdown is specific, and mRNA levels of opposing tagged TbERP constructs were unaffected, even under significant protein depletion (not shown).

We first confirmed the extent of specific knockdown of the target protein for each of the TbERP RNAi cell lines (e.g., TY-TbERP1 under TbERP1 silencing). In each case, protein levels were diminished to below detectable levels, indicating near-complete depletion of each TbERP under RNAi specific silencing (Fig. 6, diagonal from top left to bottom right). We then evaluated the remaining TbERP proteins for stability under specific RNAi silencing of each of the remaining TbERP genes in the cohort, e.g., TbERP1 expression under TbERP2 silencing. TbERP2 RNAi had the greatest effect on the observed steady-state protein levels of the other TbERPs; TbERP1, TbERP3, and TbERP8 levels were completely ablated by loss of TbERP2 (Fig. 6, column 2). Likewise, TbERP1 RNAi completely ablated TbERP3 and TbERP8 but had only a modest effect on TbERP2 (Fig. 6, column 1). Loss of TbERP8 also severely destabilized TbERP1 and TbERP3 but only modestly affected TbERP2 levels (Fig. 6, column 4). Finally, TbERP3 RNAi had a severe effect on TbERP8 levels, a modest effect on TbERP1 levels, and no detectable effect on TbERP2 levels (Fig. 6, column 3). As a control, the cultures presented in Fig. 6 were analyzed by qRT-PCR to assess the specificity of RNAi knockdown. In all cases, only the intended target mRNA was affected; all other TbERP mRNA levels were normal (data not shown). This confirms that TbERP proteins are dependent on one another for stability and support a model where TbERP1, TbERP2, TbERP3, and TbERP8 assemble and function within higher-order heterooligomeric TbERP complexes.

### TbERP expression and function in procyclic-form cells.

Procyclic-form trypanosomes express a unique major surface coat protein, procyclin (41), with a GPI anchor structure distinct from that of VSG in BSF cells (42). Given the specific expression of TbERP1, TbERP2, TbERP3, and TbERP8 during bloodstream-form growth and their function in GPI-dependent trafficking, we set out to determine if there was a TbERP cohort expressed in procyclic-form cells that also functioned in GPI-dependent transport.

The TbERP *in situ* tagging constructs were transfected into PCF cells, and expression of the fusion proteins was assessed by Western blotting with anti-TY or anti-HA antibodies. TbERP1, TbERP2, TbERP4, and TbERP8 species of the correct size were readily detectable by immunoblotting (Fig. S1A), but TbERP3, TbERP5, TbERP6, and TbERP7 were either nonexpressed or below the limit of detection in PCF cells (not shown). TbERP1 to TbERP8 mRNAs, however, were all readily detected by qRT-PCR (Fig. S2). These results suggest a specific procyclic-form cohort of TbERP proteins, TbERP1, TbERP2, TbERP4, and TbERP8, and confirm posttranscriptional regulation of TbERP protein levels.

**FIG 7:**
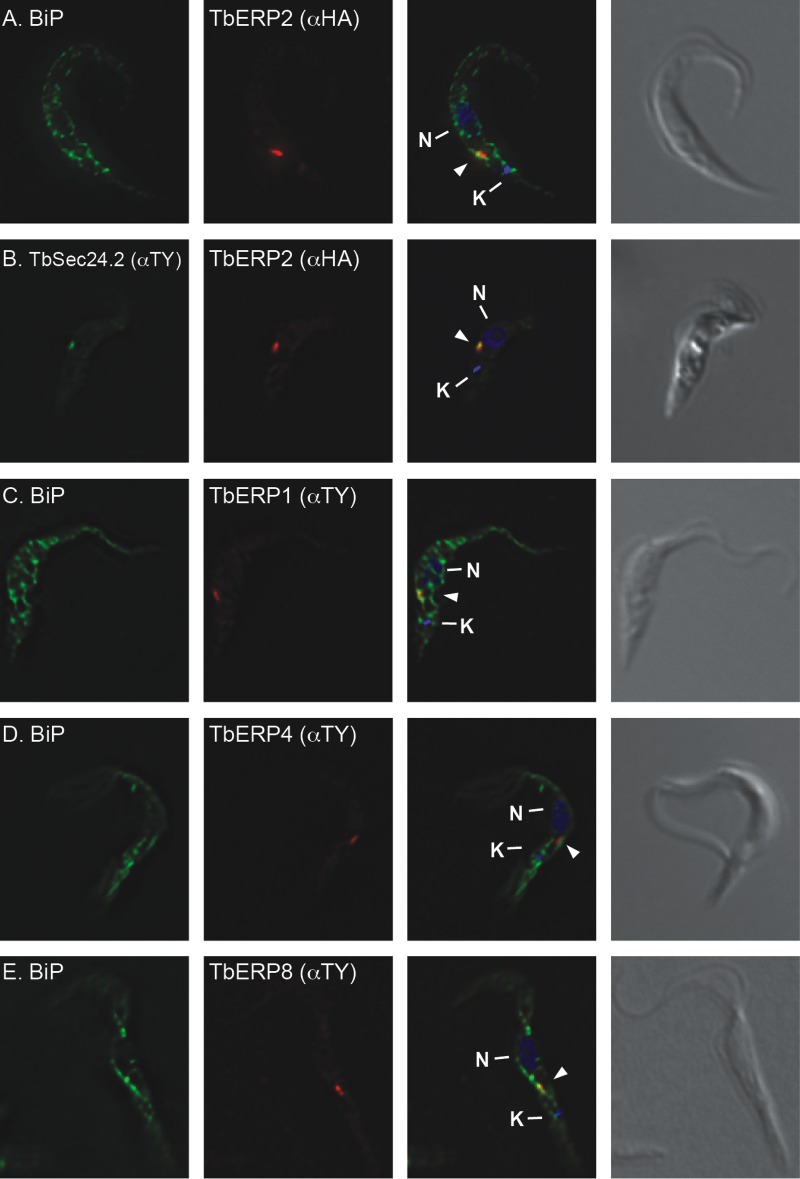
TbERP1, TbERP2, TbERP4, and TbERP8 localize to ERES in PCF cells. The *in situ* tagging constructs for TbERP2 (A), TbERP2 and TbSec24.2 (B), TbERP1 (C), TbERP4 (D), and TbERP8 (E) were transfected into the PCF cell line. Cells were fixed, permeabilized, and stained with anti-BIP, anti-TY, and/or anti-HA as indicated. Cells were then stained with appropriate fluorescently labeled secondary antibodies (BiP, green; HA, red; TY, green or red) and DAPI to visualize nucleic acid (blue). Three-channel merged images and corresponding DIC images are presented at right. All images are deconvolved sum-stack projections of representative G_1_-phase cells. Arrowheads, TbERP-positive foci; N, nucleus; K, kinetoplast.

The *in situ*-tagged PCF TbERP1, TbERP2, TbERP4, and TbERP8 cell lines were evaluated for TbERP localization by immunofluorescence microscopy. In each case, the TY- or HA-tagged TbERP protein (red) was evaluated versus the endogenous ER marker BiP (green) (Fig. 7A and C to E). Each TbERP localized to a discrete focus within the postnuclear region and overlapping the larger BiP signal, consistent with typical ERES localization in PCF cells (43, 44). To confirm ERES occupancy, the HA-TbERP2 cell line was transfected with TY-TbSec24.2. Costaining in this doubly tagged cell line revealed colocalization of TbERP2 and TbSec24.2 (Fig. 7B).

**FIG 8:**
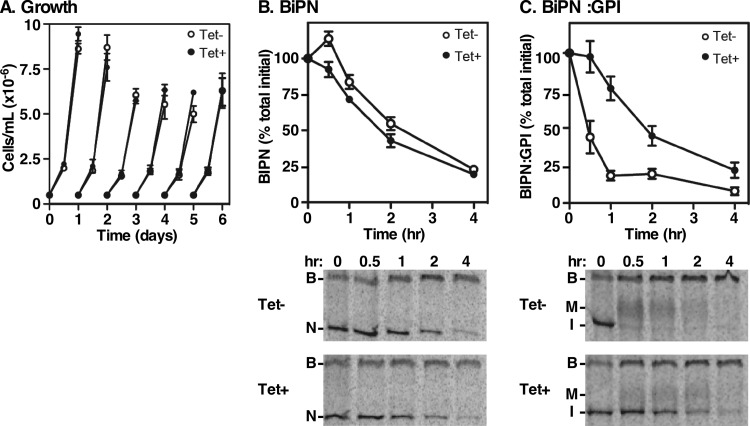
TbERP4 mediates GPI-dependent trafficking in PCF cells. (A) The tetracycline-inducible PCF TbERP4 RNAi cell line was cultured with or without tetracycline as indicated. Cell density was measured by hemacytometer every 24 h, and cells were adjusted back to the starting density (5 × 10^5^ cells/ml) every 48 h. Mean ± SEM values from triplicate cultures (for each treatment) are presented. (B and C) PCF TbERP4 RNAi cells containing the BiPN or BIPN:GPI reporters were cultured for 48 h with and without tetracycline as indicated to induce dsRNA. Cells were pulse (10 min)-chase (3 h) radiolabeled, and BiP polypeptides were specifically immunoprecipitated from whole-cell lysates at the indicated chase times and analyzed by SDS-PAGE/phosphorimaging (1 × 10^7^ cell equivalents per lane). (Top) Quantification of BiPN or BiPN:GPI normalized to *T*0 (mean ± SEM, *n* = 3). (Bottom) Representative phosphorimages of cell-associated BiP polypeptides. B, native BiP; N, BiPN; M, mature BiPN:GPI; I, immature precursor BiPN:GPI.

Among the PCF-expressed cohort of TbERPs, TbERP4 was unique relative to those expressed in BSF cells. Therefore, to evaluate its function during this growth stage, we generated a PCF RNAi cell line specifically targeting TbERP4. Following 48 h of tetracycline induction, TbERP4 mRNA levels were significantly reduced (Fig. S1C), confirming knockdown (25% ± 6% of original message remaining, *n* = 3). TbERP4 silencing, however, had no detectable effect on growth even after 12 days of induction (Fig. 8A).

In order to evaluate the role of TbERP4 in GPI-dependent trafficking in PCF trypanosomes, we transfected TbERP4 RNAi cells with the constitutive GPI-minus and GPI-plus reporters BiPN and BiPN:GPI, respectively. We then evaluated reporter trafficking rates following TbERP4 silencing. As in BSF cells, BiPN transport was measured as secretion to the medium, which was entirely unaffected by TbERP4 RNAi (Fig. 8B). BiPN:GPI transport was assessed by the conversion of the immature ER form (I) to the mature Golgi form (M) and was severely delayed under TbERP4 RNAi silencing relative to control cells (~4-fold, Fig. 8C). These results indicate that TbERP4 functions to facilitate GPI-anchored cargo transport in procyclic-form cells.

## DISCUSSION

We have presented the first analyses of p24 family proteins in *T. brucei*. All eight putative p24 proteins identified in the *T. brucei* genome display the hallmark features of p24s, including an N-terminal signal sequence, a GOLD domain, a coiled-coil domain, a transmembrane domain, and a C-terminal cytoplasmic tail containing motifs consistent with interaction with COPII for forward transport and COPI for retrograde transport. Of the eight orthologues, only four were expressed and functional during BSF growth: TbERP1, TbERP2, TbERP3, and TbERP8. During PCF growth, a different cohort was expressed: TbERP1, TbERP2, TbERP4, and TbERP8. Interestingly, mRNA corresponding to all eight TbERPs could be readily detected by qRT-PCR, at roughly similar levels, in both BSF and PCF cells. This suggests that a posttranscriptional mechanism regulates cell-type-specific expression, likely by translational control or protein stability, resulting in distinct functional TbERP cohorts. Stage-specific regulation of TbERPs mimics the more complex regulation seen in metazoans, although by a different mechanism, where p24 mRNAs as well as proteins exhibit tissue-specific expression patterns and splicing variants and are essential for specific developmental transitions (40, 45, 46). It is striking that in BSF cells the present, but nontranslated, TbERP4 to TbERP7 mRNAs were uniquely resistant to depletion by RNAi. This suggests that the mechanism of translation suppression of TbERP4 to TbERP7 mRNAs may also block degradation by the RNAi machinery. This potential interaction between translational silencing and RNAi resistance has been noted for subsets of maternal mRNAs in *Drosophila* oocytes, although precise mechanisms underlying this phenomenon remain unclear (47). A simple model to account for these coincidental phenotypes in trypanosomes might be sequestration of TbERP mRNAs from the translational and RNAi machinery, either in a discrete subcellular location (nucleus?) or in an RNA-protein particle. These possibilities will be the subject of future investigations.

The BSF cohort was evaluated for its role in secretory trafficking via RNAi silencing. Depletion of any of these TbERPs had little effect on growth; only TbERP2 ablation resulted in a modest, but significant, growth defect observable after 72 h of RNAi induction. The apparent nonessentiality of the individual TbERP genes may speak to redundancy with another, as-yet-unknown, cargo receptor(s). Alternatively, residual TbERP proteins may persist under RNAi silencing sufficient to sustain function, albeit reduced in regard to ER exit. It is worth noting, however, that the entire p24 gene family in yeast is nonessential; an eight-way p24-knockout strain is viable and grows normally under unstressed laboratory conditions (48). Additionally, yeast p24 proteins show some compensatory behavior in deletion strains, with compensatory pairs being members of the same p24 subclass, e.g., those that show greater protein homology (28, 49).

The transport defect seen for GPI-anchored cargo under silencing of each member of the BSF cohort suggests specific interaction(s) of one or more TbERP proteins, likely in a complex (see below), with the GPI anchor, consistent with known p24 function in yeast and mammalian cells (28, 30, 31, 50). Importantly, the decreased rate of BiPN:GPI transport under TbERP RNAi was essentially the same as the normal rate for BiPN with no GPI at all. Thus, it appears that the BSF TbERP cohort facilitates a general GPI-dependent acceleration of ER exit. Silencing of the obligate heterodimeric COPII subunits TbSec23.2 and TbSec24.1 resulted in a similar GPI dependence—BiPN:GPI trafficking was retarded, whereas BiPN was not (22). Collectively, these data support a model in which TbERPs function as adaptors between the GPI anchor (luminal) and the TbSec23.2/TbSec24.1 heterodimer (cytoplasmic) in order to facilitate specific incorporation of GPI cargo into actively budding COPII vesicles at ERES.

The BSF cohort of TbERPs demonstrated extensive interdependence for expression—silencing of any one TbERP severely impacted the steady-state levels of the others. This effect is clearly posttranscriptional as in each case only the targeted mRNA was depleted. Reduced levels of the “bystander” TbERPs could be due to translational repression and/or increased turnover. Within the cohort, there is a clear hierarchy of interdependence. TbERP2 is dominant in that its depletion completely eliminated all the other TbERPs. Thus, it is likely not a coincidence that only TbERP2 is required for normal growth. TbERP1 is next in that its loss completely ablated TbERP3 and TbERP8 but only modestly affected TbERP2. Last are TbERP3 and TbERP8, the loss of each of which is completely reciprocated by the other but had no effect on TbERP2 and only partially affected TbERP1. This hierarchy of interdependence and phenocopying of TbERPs for GPI-dependent ER exit are reminiscent of the situation in yeast (28, 30, 31) and are highly suggestive of TbERP heterooligomers. *Saccharomyces* p24 genes that phenocopied for GPI trafficking when deleted (Emp24, Erv25, Erp1, and Erp2) were predictive of physically interacting p24 subunits (25, 28, 30, 48). Likewise, mammalian p24 subunits that exhibited similar trafficking defects when depleted were found to physically interact (26, 31, 50, 51). Strikingly, the centrality of TbERP2 in trypanosomes is similar to Emp24 in *S. cerevisiae*, loss of which completely destabilizes the other subunits (Erv25, Erp1, and Erp2), while the reverse depletions show minimal to no effect on Emp24 levels (28). Yet, as in trypanosomes, four p24s are required for efficient ER exit of GPI cargo.

Given the parallels to the yeast system, and in keeping with the concept that p24s assemble in combinatorial ways to generate cargo receptors, two broad scenarios can be envisioned in trypanosomes. Each accommodates the apparent primacy of TbERP2, which is also supported by the synergistic toxicity that we observe between TbERP2 depletion and constitutive BiPN:GPI overexpression. Each scenario, however, requires that a subset of TbERP oligomers contains the entire cohort in order to account for the phenocopying in acceleration of GPI-dependent ER exit. In the first scenario, there is a single fixed oligomer that includes all four members of the BSF TbERP cohort (no specific stoichiometry implied). Within this complex, TbERP2 is “dominant” such that loss of the other “lesser” TbERPs only partially impacts TbERP2, but its loss is catastrophic for stability of the entire complex. In this scenario, generation of multiple cargo specificities, e.g., GPI versus TbCatL (see below), might be controlled by altering the internal stoichiometry of the complex. Alternatively, there may be multiple complexes, all containing TbERP2 but having variable complements of the other TbERPs (again, no specific stoichiometry implied). Loss of TbERP2 would impact all the complexes, while loss of any of the others would only impact those TbERPs in the same complex. Since not all complexes contain all TbERPs, other than TbERP2, the effect of ablating any one would only be partial on the others. Again, in this scenario, varying the component stoichiometry of the complexes could account for phenocopying for different classes of cargo (see below for discussion of TbCatL). We have made extensive attempts to directly demonstrate TbERP-TbERP interactions (as well as TbERP-COPII and TbERP-cargo interactions) via a standard panel of biochemical techniques; unfortunately, none have been successful to date. Nevertheless, it is very likely, based on our results and on precedents in other systems, that TbERP1, TbERP2, TbERP3, and TbERP8 associate in functional heterooligomers and that an oligomer comprised of at least one subunit of each is the minimal assembly required for GPI-dependent trafficking from the ER.

Transport of the soluble cathepsin L orthologue, TbCatL, is also impaired (2- to 4-fold) by TbERP knockdown and phenocopies across the entire BSF cohort. TbCatL has a forward trafficking rate that is greatly increased relative to bulk flow (*t*_1/2_ of ~15 min versus ~90 min for BiPN), consistent with forward trafficking signals that facilitate ER exit. These results suggest that TbERPs also mediate recruitment of TbCatL to COPII vesicles. This is not without precedent, as transport of non-GPI-anchored invertase is sensitive to knockout of p24s in yeast (25, 34). All of the general issues outlined above for the role of TbERP complexes in GPI-dependent ER exit, particularly the need for all four BSF TbERPs to account for phenocopying, would apply to TbCatL recruitment, with two exceptions. First, unlike the ER exit of GPI cargo, TbCatL is handled redundantly by both the TbSec23.2/TbSec24.1 and TbSec23.1/TbSec24.2 heterodimers (22). Second, loss of TbERP3 has less effect on TbCatL trafficking than does loss of the other TbERPs (2-fold versus 4-fold), whereas its loss has similar effects on GPI-dependent ER exit. These data could be explained by the existence of complexes containing all four TbERPs but with different TbERP3 stoichiometries that influence specific interactions with cargo and COPII machinery. It is also possible that the effect of TbERP depletion on TbCatL is indirect, but the fact that bulk flow reporter BiPN is not affected argues against this.

A distinct cohort of TbERPs is expressed in procyclic trypanosomes: TbERP1, TbERP2, TbERP4, and TbERP8. As mentioned above, mRNAs for all eight TbERPs are found in both life cycle stages, suggesting possible stage-specific regulation by translational control. This issue is under investigation. As in BSF trypanosomes, the PCF cohort colocalizes to ERES, and knockdown of the unique component, TbERP4, negatively impacts GPI-dependent ER exit. The use of different TbERP cohorts for GPI-dependent ER exit may reflect stage-specific structural differences in the glycan core of protein-bound GPI anchors. Indeed, the remodeled glycan core of mature GPI anchors in the ER is the moiety recognized by p24 complexes in both yeast and mammalian cells (30, 31). In BSF trypanosomes, a variable number of galactose residues may be added to each residue of the trimannosyl core after transfer of the GPI precursor to VSG. This process begins in the ER and continues during transit of the Golgi compartment (52–54). In contrast, PCF trypanosomes express a different GPI-anchored surface protein, procyclin (41). Its GPI anchor has a branched poly-*N*-acetyllactosamine side chain that begins with attachment of a digalactose moiety to Man2 of the GPI core (7). Whether this is initiated in the ER is not known, but stage-specific differences in mature ER GPI glycan structures clearly exist. One caveat to this scenario is that the GPI core structure on a subclass of VSGs is not modified by galactosylation (54). However, there are also stage-specific differences in GPI lipid configurations. BSF trypanosomes have a dimyristoyl glycerol anchor while PCF trypanosomes have lysoacylglycerol with direct fatty acylation of the 2 position of inositol (55). There is precedent for p24-lipid interactions, albeit with sphingolipids, and a similar phenomenon may occur in trypanosomes (26). In either case, TbERP3 and TbERP4 may serve as interchangeable elements to accommodate these differences. Interestingly, TbERP3 and TbERP4 are the most closely related of the eight TbERPs (74.5% similarity, 31.0% identity), which may account for their ability to substitute for each other among the three other invariant subunits.

The external surface of trypanosomes is covered by a uniform coat composed of a single GPI-anchor protein, VSG in BSF and procyclin in PCF. It is not surprising, given the abundance of these proteins (0.5 × 10^7^ to 1 × 10^7^ copies per cell), that GPI anchor structure and biosynthesis were first characterized in trypanosomes (reviewed in reference 56). VSG is the most critical virulence factor for pathogenesis in the mammalian host. Similarly, the procyclin coat increases parasite resistance to acid protease digestion in the fly midgut, allowing for efficient development and successful transmission (57, 58). ER exit of these cargoes is GPI dependent, with VSG being selectively trafficked by a subset of the TbCOPII machinery in BSF trypanosomes (9–11, 22). Our work here clearly defines trypanosomal p24s as the adaptor between these two processes—cargo recognition in the lumen of the ER and recognition of COPII on the cytoplasmic face of ERES. An unexpected finding is the posttranscriptional developmental regulation of TbERP expression and function, raising questions about the mode of regulation (translation and/or protein stability) and whether unique interactions exist between the GPI-anchored cargo and COPII machinery in PCF trypanosomes. The cell-type-specific adaptations in *T. brucei* that accommodate the GPI-protein burden in differing life stages will be the focus of future efforts, in order to more fully understand the role of GPI-anchored proteins essential to the parasite life cycle.

## MATERIALS AND METHODS

### Maintenance of trypanosomes.

Bloodstream-form *Trypanosoma brucei brucei* cells (Lister 427 strain, expressing VSG221) were cultured in HMI-9 medium supplemented with 10% fetal bovine serum (FBS) and 10% Serum Plus (SAFC Biosciences, Lenexa, KS) at 37°C with 5% CO_2_ (59). The single-marker Lister 427 strain (60) used for conditional induction of dsRNA was maintained in HMI-9 supplemented with 20% tetracycline-free fetal bovine serum (Atlanta Biologicals, Lawrenceville, GA). In all BSF experiments, cells were harvested at mid- to late log phase (0.5 × 10^6^ to 1 × 10^6^ cells/ml). Cultured procyclic-form 29-13 cells (derived from strain 427) were cultured in Cunningham’s medium (61) supplemented with 10% fetal bovine serum at 27°C at ambient CO_2_. Cells used for conditional induction of dsRNA were maintained in Cunningham’s medium supplemented with 10% tetracycline-free fetal bovine serum (Atlanta Biologicals, Lawrenceville, GA). For all PCF experiments, cells were harvested at mid- to late log phase (0.5 × 10^7^ to 1 × 10^7^ cells/ml).

### Construction of epitope-tagged and RNAi cell lines.

For construction of all RNAi plasmids, a 400- to 600-bp fragment of each TbERP open reading frame (ORF) was amplified with primers containing nested terminal restriction sites: 5′ BamHI/XhoI and 3′ XbaI/AscI (for TbERP2, TbERP3, TbERP4, and TbERP8) or 5′ NdeI/XhoI and 3′ XbaI/AscI (for TbERP1). See Table S1 in the supplemental material for specific RNAi targeting sequences. Restriction-digested PCR products were sequentially inserted in opposing orientations into the pLEW100.v5X:Pex11 stem-loop vector (39), to generate tetracycline-inducible stem-loop dsRNA targeting each TbERP gene. Constructs were linearized with NotI for transfection, and expression of the RNAi stem-loop was induced with 1 µg/ml tetracycline. The BiPN and BiPN:GPI reporters (in pXS5^pac^) have been described previously (10, 37). Plasmids were linearized for transfection with XhoI.

For localization experiments, TbERP2 was *in situ* epitope tagged with three tandem repeats of the hemagglutinin (HA) tag (YPYDVPDYA), and TbERP1, TbERP3, TbERP4, and TbERP8 were *in situ* epitope tagged with a single TY epitope tag (EVHTNQDPDL) by homologous recombination at the 5′ end of each gene. This strategy allows for detection of the tagged gene at levels approximating wild-type expression. The tagging vector, a derivative of pXS5 (with either a puromycin or a hygromycin resistance cassette) (62), has the EP1 procyclin signal sequence (Tb927.10.20160, nucleotides [nt] 1 to 111) fused in frame to either the 3×HA or 1×TY epitope tag and positioned downstream of the βα-tubulin intergenic region. Fragments corresponding to each entire TbERP open reading frame (ORF), minus the native signal sequence predicted by SignalP (63), were PCR amplified and inserted in frame and downstream of the epitope tag, using 5′ NheI and 3′ MfeI restriction sites. Inserts were TbERP1 (nt 115 to 687), TbERP2 (nt 96 to 660), TbERP3 (nt 76 to 618), TbERP4 (nt 66 to 613), TbERP5 (nt 94 to 699), TbERP6 (nt 106 to 678), TbERP7 (nt 94 to 744), and TbERP8 (nt 97 to 762). An ~500-bp segment immediately upstream of the 5′ end of each corresponding TbERP ORF was PCR amplified and inserted into the expression vector upstream of the resistance cassette using XmaI-AscI. Each completed construct contains (5′ to 3′) a gene-specific 5′ upstream flanking region, antibiotic resistance cassette, the βα-tubulin intergenic region, EP1 start codon and signal sequence (nt 1 to 207), and an in-frame epitope tag (3×HA or 1×TY), followed by the corresponding TbERP ORF, beginning just downstream of the endogenous signal peptide. Duplicate tagging constructs were prepared with hygromycin and puromycin cassettes to allow tagging of both chromosomal alleles. The *in situ* tagging constructs were excised for transfection with XmaI/MfeI and stably transfected into wild-type or TbERP RNAi cell lines. To generate tetracycline-inducible epitope-tagged TbERP4 to TbERP7 reporters, the fusion construct was amplified from the *in situ* vectors and inserted into the tetracycline-responsive expression vector pLEW100x using HindIII-BamHI (39). The TbSec24.1-TY and TbSec24.2-TY *in situ* tagging vectors were published previously (22). Clonal transfectants were selected with hygromycin or puromycin as appropriate.

Genomic DNA from Lister 427 cells was used as the template for all PCR amplifications. All dsRNA plasmids and epitope-tagging vectors were sequence verified prior to transfection. Genotypes for *in situ*-tagged cell lines were verified by PCR (data not shown). Linearized constructs were introduced into appropriate host *T. brucei* cells using Amaxa nucleofection as described elsewhere (64). Stable clonal transfectants were isolated by limiting dilution under appropriate antibiotic selection.

### Antibodies, secondary antibodies, and blotting reagents.

The rabbit anti-VSG221, rabbit anti-BiP, rabbit anti-TbCatL, and rabbit anti-TbHsp70 antibodies have been described previously (12, 62, 65, 66). The mouse anti-TY (UAB Hybridoma Facility, Birmingham, AL) was used as described in previous reports (38). The rabbit anti-HA (Sigma-Aldrich, St. Louis, MO), rat anti-HA (Roche), Alexa Fluor (Molecular Probes), and horseradish peroxidase (HRP)-conjugated (Sigma-Aldrich, St. Louis, MO) goat secondary antibodies were used according to manufacturer’s recommendations.

### Metabolic radiolabeling and immunoprecipitation.

Pulse-chase radiolabeling of trypanosomes with [^35^S]methionine-cysteine (PerkinElmer, Waltham, MA) and subsequent immunoprecipitation of secretory reporters were conducted as described previously (62, 66). VSG transport kinetics were determined via the established assay (35, 38). Briefly, following a short pulse of metabolic radiolabeling, cells are lysed in water at the indicated time points. This exposes membrane-bound, surface VSG to conversion by endogenous GPI-phospholipase C. The newly soluble VSG is separated from the cell-associated VSG by centrifugation, and the resulting fractions are solubilized and immunoprecipitated under standard conditions. Pulse times were 2 min (VSG), 5 min (BiPN:GPI reporter), and 10 min (TbCatL and BiPN reporters). Chase times are indicated in the figures. Lysates for immunoprecipitation were prepared in radioimmunoprecipitation assay (RIPA) buffer (50 mM Tris-HCl, pH 8.0, 150 mM NaCl, 1.0% NP-40, 0.5% deoxycholate, and 0.1% SDS). Precipitated material was fractionated by SDS-polyacrylamide gel electrophoresis (PAGE) and visualized with the Typhoon phosphorimaging system (GE Healthcare). Band intensities were quantified using the ImageQuant software packages as described previously (67).

### RNA extraction and qRT-PCR.

Total RNA was extracted from log-phase cells using the RNeasy minikit (Qiagen, Valencia, CA) with on-column DNase I digestion, both according to the manufacturer’s instructions. Corresponding cDNA was generated using 1 µg total RNA and the iScript cDNA synthesis kit (Bio-Rad, Hercules, CA), according to the manufacturer’s instructions. cDNAs were diluted and used in PCR mixtures containing Power SYBR green master mix (Life Technologies, Inc.) and 0.5 µM (each) forward and reverse primer. Target sequences were TbERP1 nt 13 to 115, TbERP2 nt 158 to 266, TbERP3 nt 233 to 351, TbERP4 nt 6 to 116, TbERP5 nt 149 to 249, TbERP6 nt 566 to 649, TbERP7 nt 10 to 102, TbERP8 nt 177 to 293, and TbZFP3 (Tb927.3.720) nt 226 to 292. Quantitative real-time PCR (qRT-PCR) was performed using the Applied Biosystems Step-One real-time system. Specific TbERP mRNA levels were determined relative to the internal reference gene TbZFP3 (68) in biological triplicate (means ± standard errors of the means [SEM]).

### Immunofluorescence microscopy.

Log-phase BSF parasites were fixed in phosphate-buffered saline (PBS) with 2% formaldehyde and allowed to settle onto SuperFrost Plus microscopy slides (Fisher Scientific) (plain glass slides for log-phase PCF cells), for 30 min at room temperature as described previously (38). Fixative was removed, and cells were permeabilized for 30 min in PBS–0.5% NP-40 (30 min, room temperature) and then blocked for 30 min in blocking solution (PBS, 0.1% NP-40, 10% goat serum). Cells were stained with primary antibodies diluted in blocking solution, washed, and then stained with the appropriate secondary antibodies and 4′,6-diamidino-2-phenylindole (DAPI), also in blocking solution. Slides were washed and then mounted in PBS-glycerol (1:1) for visualization. Serial image stacks (0.2-µm Z-increment) were collected with capture times from 100 to 500 ms (100× PlanApo, oil immersion, 1.46 numerical aperture [NA]) on a motorized Zeiss AxioImager M2 equipped with a rear-mounted excitation filter wheel, a triple pass (DAPI/fluorescein isothiocyanate [FITC]/Texas Red) emission cube, differential interference contrast (DIC) optics, and an Orca AG charge-coupled device (CCD) camera (Hamamatsu, Bridgewater, NJ). All images were collected with Velocity 6.0 acquisition software (Improvision, Lexington, MA), and individual channel stacks were deconvolved by a constrained iterative algorithm, pseudocolored, and merged using Velocity 6.0 restoration software. All images presented are summed-stack projections of individual or merged channels. The *xyz* pixel precision of this arrangement has been validated in reference 22 (see Fig. S1 there).

### TbERP Western blotting under RNAi silencing.

Transgenic cell lines expressing the tetracycline-inducible RNAi stem-loop constructs as well as the epitope-tagged TbERP gene of interest were grown with or without tetracycline for 72 h (in triplicate). Whole-cell lysates were separated by SDS-PAGE (1 × 10^7^ cell equivalents/lane) and transferred to an Immobilon-P membrane (Millipore Corporation) using the Owl semidry apparatus (Thermo Fisher Scientific). Membranes were blocked (5% milk, 1% goat serum) and then incubated with mouse anti-TY (1:250) or rat anti-HA (1:500) for 1 h. Blots were stripped and reprobed with rabbit anti-Hsp70 (1:8,000). Blots were visualized on the ChemiDoc XRS+ Western imaging system (Bio-Rad, Hercules, CA) using the Pierce SuperSignal West Pico chemiluminescent substrate (Thermo Fisher Scientific).

### Statistical analyses.

All statistical tests were conducted in the GraphPad Prism 5 software package. Replicates are indicated in the figures.
