# Supplementary material for: Life Stage-Specific Cargo Receptors Facilitate Glycosylphosphatidylinositol-Anchored Surface Coat Protein Transport in Trypanosoma brucei
Source: mSphere. 2017 Jul 12;2(4):e00282-17. doi: 10.1128/mSphere.00282-17 (PMC5506558; doi:10.1128/mSphere.00282-17)
Supplement: TABLE S1 [file sph004172321st3.pdf]

Table S1: *Trypanosoma brucei* p24 orthologues

Name	TriTryp Accession	CDS length (nt)	RNAi target (nt)	C term peptide
TbERP1	Tb927.11.15230	687	31-471	RRIFN*KRRVV*
TbERP2	Tb927.9.15090	660	63-556	EMWHLKRYFR*KKRLID*
TbERP3	Tb927.8.8030	618	8-500	KRFLE*RKSFV*
TbERP4	Tb927.4.4350	612	1-612	KCFLE*RKSFV*
TbERP5	Tb927.10.9270	699	53-523	SEKYLERFFI*KQKIA*
TbERP6	Tb927.7.3600	678	205-269	KRMFNRKGTRAVA
TbERP7	Tb927.8.7280	744	126-662	RTLRFLL*RCKPQ*
TbERP8	Tb927.10.6640	762	55-613	RLLKSTLRE*KKLV*

Putative targeting signals: COPI, KXKXX (italicized); COPII, di-hydrophobic (underlined)
