# Supplementary figures and images for: Life Stage-Specific Cargo Receptors Facilitate Glycosylphosphatidylinositol-Anchored Surface Coat Protein Transport in Trypanosoma brucei
Source: mSphere. 2017 Jul 12;2(4):e00282-17. doi: 10.1128/mSphere.00282-17 (PMC5506558; doi:10.1128/mSphere.00282-17)

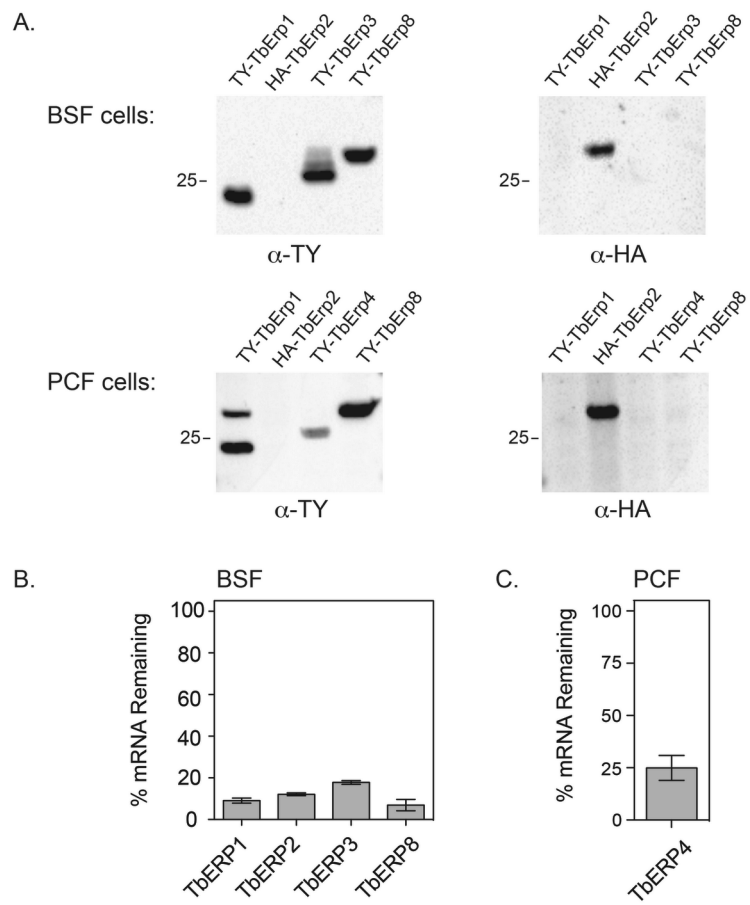

**Fig. S1**

Supplement: FIG S1 [file sph004172321sf1.pdf]

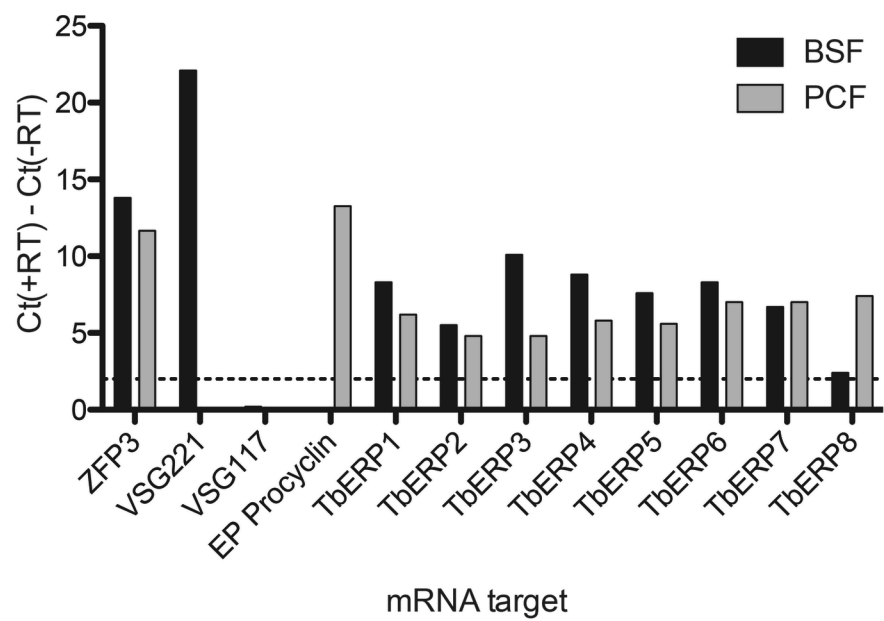

**Fig. S2**

Supplement: FIG S2 [file sph004172321sf2.pdf]
